# WORKPLACE AND PERSONAL ATTRIBUTES AFFECTING WORKPLACE PSYCHOLOGICAL BURDEN OF MATURE AND AGING WORKERS IN HONG KONG

**DOI:** 10.1093/geroni/igad104.2163

**Published:** 2023-12-21

**Authors:** Daniel W L Lai, Xue Bai, Vincent W P Lee, Kin Cheung, Calvin W Luk, Chi-Ko Lee

**Affiliations:** Hong Kong Baptist University, Hong Kong, Hong Kong; The Hong Kong Polytechnic University, Kowloon, Hong Kong; Hong Kong Baptist University, Hong Kong, Hong Kong; The Hong Kong Polytechnic University, Kowloon, Hong Kong; The Hong Kong Polytechnic University, Kowloon, Hong Kong; The Hong Kong Polytechnic University, Kowloon, Hong Kong

## Abstract

Older employees are valuable human resources for society. However, changes in cognitive abilities and mental health in older persons may affect their ability to work, as well as their safety and health at work. The purpose of this study is to investigate the effects of older workers’ personal attributes such as demographics and lifestyle, physical health status, mental health status, labour intensity of employment, and experience of occupational and health risks on their psychological burden at work. A quantitative telephone survey was conducted with a stratified random sample of 1,225 workers aged 40 or above. The results of multiple linear regression analysis indicated that the men and workers with better sleep quality tended to suffer less from psychological burden at work. Older workers with longer weekly work hours and incidents of work-related injuries tended to have a higher level of psychological burden, whereas a safer work environment perceived by the older workers was associated with a lower psychological burden. To accommodate their needs, employers are recommended to allow the older employees choices of reducing work hours and flexible work arrangements. The application of assistive devices and relevant technologies for minimizing the threats to safety and health for the sake of the employees’ long-term psychological well-being is important for institutions and employers to consider.

